# A Splenic Rupture Following a Singleton Spontaneous Vaginal Delivery: The First-Known Case Report in Saudi Arabia

**DOI:** 10.7759/cureus.58246

**Published:** 2024-04-14

**Authors:** Ruqayyah A Ahmed, Faten A Yaseen, Reem S Aljudaibi, Nabata A Ahmed

**Affiliations:** 1 Department of General Medicine and Surgery, Batterjee Medical College for Science and Technology, Jeddah, SAU; 2 Department of Obstetrics and Gynecology, Aya Specialist Hospital, Jeddah, SAU; 3 Department of Obstetrics and Gynecology, Saudi Board in Obstetrics and Gynecology, Aya Specialist Hospital, Jeddah, SAU

**Keywords:** emergency laparotomy, postpartum complication, splenectomy postpartum, acute abdominal pain, s: postpartum hemorrhage, normal spontaneous vaginal delivery (nsvd), spontaneous splenic rupture

## Abstract

A spontaneous rupture of the spleen during pregnancy or post-delivery is an exceptionally rare but potentially fatal maternal complication that poses a significant challenge in diagnosis and management. Herewith, we present a case of a 31-year-old female patient who experienced a spontaneous splenic rupture following a singleton vaginal delivery. Despite lacking any history of trauma or a predisposing factor, she developed symptoms of dizziness and postpartum abdominal pain, progressing rapidly to unconsciousness. Urgent abdominal ultrasound revealed significant intraperitoneal bleeding with a large peri-splenic hematoma, necessitating emergency exploratory laparotomy. Histopathological examination of a frozen section ruled out malignancy, and thus, a complete splenectomy was done, which confirmed the diagnosis of spontaneous splenic rupture. This case emphasizes the importance of close monitoring of all postpartum women, even those with low-risk pregnancies, for the early detection of any complication. Healthcare providers should maintain a high index of suspicion for rare but potentially life-threatening events to ensure timely intervention and optimal outcomes.

## Introduction

Antepartum and postpartum hemorrhages are currently the leading cause of maternal mortality and long-term morbidity worldwide. It may be due to either medical, surgical, or obstetrical factors [1]. A spontaneous or non-traumatic splenic rupture is a rare occurrence in the setting of a healthy spleen during pregnancy [2]. It is defined to occur in the absence of any associated splenic trauma, systemic disease, and absent evidence related to splenic adhesions [1]. Due to its nonspecific symptoms following delivery; the diagnosis is sometimes confusing and subsequently delayed, which can lead to adverse outcomes [3]. The lethal injury caused by splenic rupture could occur with any type of trauma, with or without predisposing factors. However, the mechanism and cause of injury in some cases is multifactorial and unclear. Several authors have considered that pregnancy itself is a risk factor for splenic rupture, which is explained by hypovolemia and alteration in abdominal organs caused by pregnancy changes [1,4]. Another cause of splenic rupture is iatrogenic, which involves involuntary damage to the spleen during a surgical operation or intervention such as performing fundal pressure (Kristeller maneuver) during the second stage of labor. Intraoperative bleeding originating from the spleen is an extremely rare complication following cesarian section operations although it can increase the risk of maternal mortality and morbidity significantly. Maternal mortality from splenic rupture is reported in a range between 0% and 45% [5]. In this case report, we present a case of spontaneous splenic rupture and intrabdominal massive bleeding following a singleton spontaneous vaginal delivery.

## Case presentation

Case presentation

**Figure 1:**
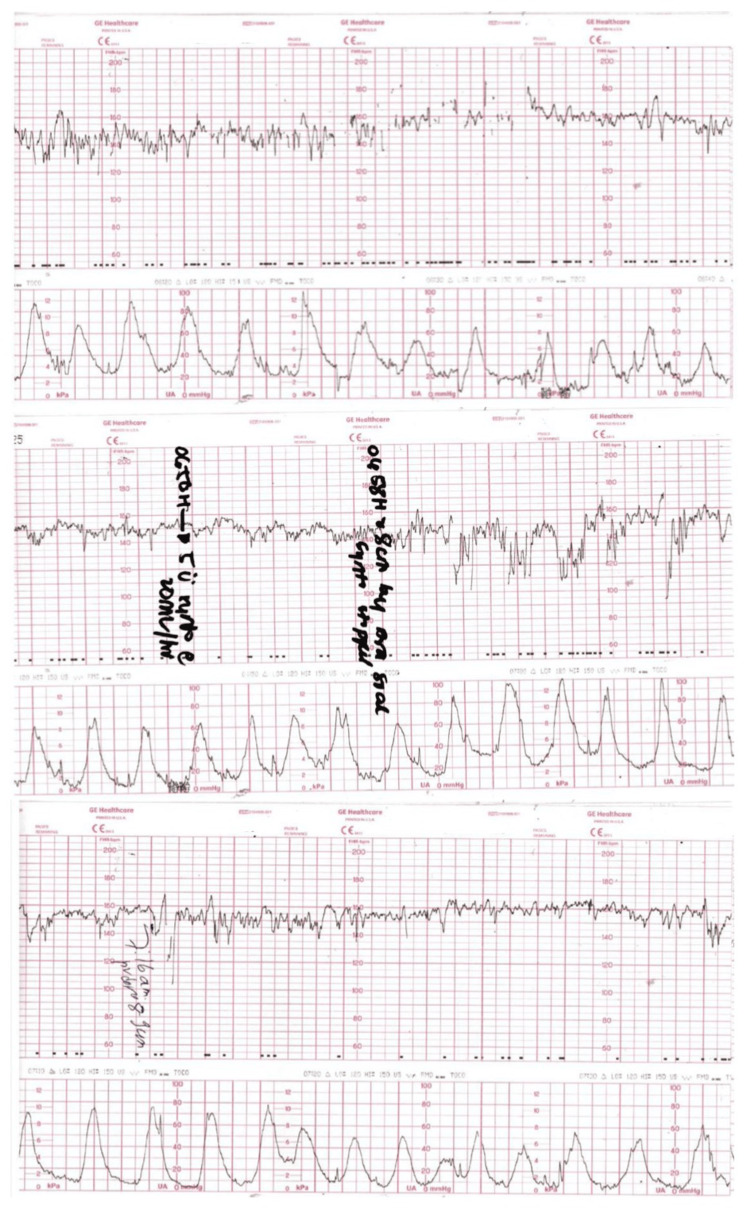
Fetal CTG CTG: cardiotocography

**Figure 2:**
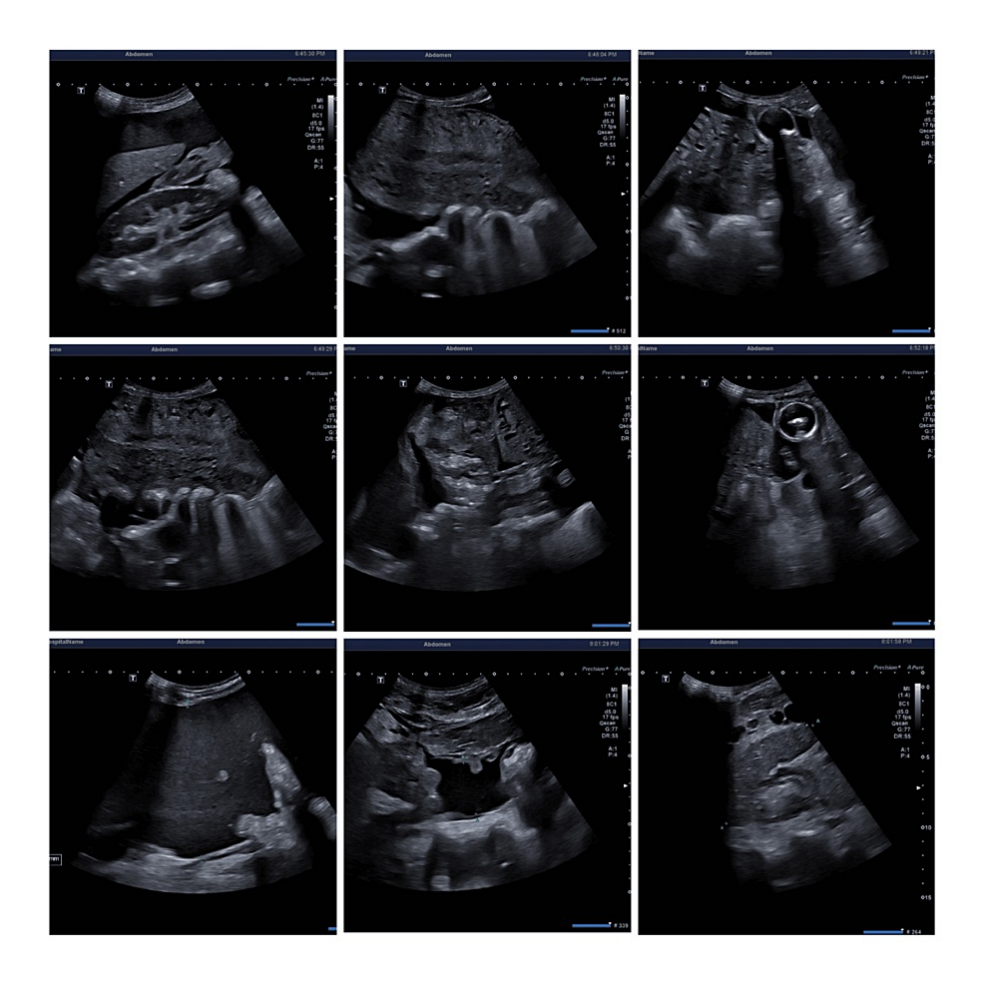
Ultrasound images of the abdomen

A 31-year-old woman, gravida 3, para 2, presented to the clinic with a gestational age of 37 weeks and 6 days calculated based on her last menstrual period. She complained of labor pain with no bleeding per vagina and adequate fetal movements. There were no significant findings in her past or family history. She was also surgically free. Her obstetric history indicated two prior successful vaginal deliveries, with the latter four years ago. She was admitted for spontaneous vaginal delivery and her cardiotocography (CTG) demonstrated a reactive trace with adequate regular contractions. She requested epidural analgesia for pain relief. Upon physical examination, the patient had a soft lax abdomen with no tenderness. Her blood pressure was recorded to be 110/70 mmHg, heart rate 74/min, and SPO_2_ 99% at room air. On pelvic examination, the cervical dilatation was 6 -7 cm. Figure 1 demonstrates the attached CTG, which showed regular contractions and recorded the fetal heart rate to be 140 to 150 bpm. The patient had a successful vaginal delivery, and she delivered a healthy male infant. Episiotomy was done and repaired, and the patient was admitted to the ward for observation. Table 1 lists the laboratory results before delivery. Following delivery, the patient was stable with a contracted uterus, mild lochia, and mild post-partum pain. Approximately eight hours following childbirth, the patient started complaining of dizziness and abdominal pain and was given a paracetamol injection of 1 gm through the intravenous route. Her dizziness continued, and she suddenly lost consciousness. The patient was resuscitated and her blood pressure reading was 80/55 and her heart rate was 99/min. She was transferred to the ICU and started on IV fluids and cardiac monitoring. Urgent pelvic abdominal ultrasound was ordered revealing a marked amount of free intraperitoneal turbid fluid collection as well as a large peri-splenic (15x8 cm) hematoma under the left hemidiaphragm around the spleen. Figure 2 displays the abdominal ultrasound images. The liver was of average size with no focal hepatic lesions or evidence of hepatic duct dilatation. The remaining organs, including the gallbladder, head of the pancreas, kidneys, ovaries, urinary bladder, and spleen, demonstrated no abnormalities under ultrasound. The uterus showed average endometrial thickness with no myometrial lesions. No source of the large bleeding could be identified. The patient became restless and developed tachycardia and severe hypotension. She was immediately transferred to the OR as an emergency case for abdominal exploration laparotomy. A transfusion of packed RBCs and fresh frozen plasma was started, along with ringer lactate IV fluids. Open laparotomy was initiated and exploration revealed splenic rupture and massive bleeding was noted. A general surgeon was called and a splenectomy was done followed by packing and closure of the abdomen. A large frozen section biopsy of the spleen was sent for histopathology, demonstrating a congestive red pulp with no evidence of malignancy. The histopathology report explained the microscopic description of the spleen as expansion of the red pulp with congestion of the blood and atrophy of the white pulp. In the hilum, three reactive lymph nodes were identified. No infarction, fibrosis, calcification, atypical cells, or malignancy was seen. The gross description of the specimen consisting of a complete splenectomy reported weight of 125.1 grams and measuring 10.0 x 7.0 x 3.3 cm with a smooth outer surface. The capsule was intact and smooth, on sectioning, the spleen showed a homogenous cut surface with blood congestion. No infarction, fibrosis, calcification, or tumor was identified grossly. Representative sections were submitted in five cassettes: A,C - fatty tissue at the hilum and D,E - Spleen tissue. Once the procedure ended, the patient was transferred to an ICU in a higher advanced facility tertiary care hospital for further monitoring and follow-up.

**Table 1:** Laboratory results

Test	Normal values	Result
Complete Blood Count (CBC), with differentials		
Erythrocyte Count	3.9 – 5.03	3.01 10^6^/uL
Hemoglobin	12 – 15.5	8.8 g/dL
Hematocrit	34.9 – 44.5	26.4 %
Platelet Count	150 - 450	4 10^3^/uL
Special Chemistry		
Bilirubin (Direct)	0.0 – 0.30	0.4 mg/dL
Bilirubin (Total)	0.2 – 1.3	0.6 mg/dL
Albumin	3.5 – 5.0	1.1 g/dL
Aspartate aminotransferase (AST)	14.36	25 U/L
Alanine transaminase (ALT)	9.52	24 U/L
Antibody Screening		
Type I, II, and III Cells	Negative	
Hepatitis B Surface Antigen	Non-reactive	
Hepatitis C Antibodies	Non-reactive	
Human Immunodeficiency Virus (HIV) Antibody/Antigen	Non-reactive	

Literature review 

Table 2 lists previous case reports with a similar presentation to our case and diagnosed as postpartum splenic rupture.

**Table 2:** Past studies reporting cases of postpartum splenic rupture

Author	YOP	Age & GPA	Mode of Delivery	Chief Complaint	Complication	Radiological or Clinical Findings	Treatment
D. Socolov, et al. [4]	2016	23, Nulliparous	Vaginal Delivery	Significant Vaginal Bleeding	One cm superficial capsular laceration, two cm above the splenic pedicle	Abdominal ultrasound - discovery of splenic rupture, indicated the presence of a splenic hematoma of 73.05 cm3 volume	Successful conservation of the spleen
Bahareh Hamedi, et al. [6]	2013	21 Years, Primigravida	Cesarian Delivery	Coffee ground vomiting	2.5×3 cm defect in splenic capsule with active bleeding from the ruptured site	Clinical evidence of free fluid was present and the blood pressure fell to 90/60 mmHg	Emergency laparotomy
Franco Londero, et al. [7]	2000	35 Years, Primigravida	Vacuum Assisted Delivery	Short strong pain in the left hypochondrium	Hypovolemic shock	Tomography revealed the presence of blood in the abdomen	Splenectomy
Ioannis Korkontzelos, et al. [1]	2023	28, Gravida 3, para 1	Elective Cesarian Section	Not mentioned	Continuous minor bleeding coming from the upper left abdomen was ascertained	Clinical evidence of splenic trauma at the inferior pole of the spleen	Conservative technique of packing, topical surgical hemostatic agents, and non-absorbable sutures
Batool Teimoori, et al. [8]	2018	33, Gravida3, para 2	Elective Cesarian Section	Dyspnea and anuria	Free intraperitoneal fluid was found in Morison’s pouch in patients with suspected incisional scar hemorrhage	Abdominal ultrasound showed: free fluid in Morison’s pouch on the bedside predicts the need for operative intervention	Splenectomy
Leah Mayne, et al. [9]	2021	29, Gravida 2, para 1	Elective Cesarian Section	Syncope, tachycardia, tender abdomen	Hypotension, intraabdominal bleeding	Abdominal/pelvic computed tomography angiogram revealed moderate hemoperitoneum	Successful conservation of the spleen
Manato Fujii, et al. [10]	2020	40, Gravida 1, para 0	Emergency Cesarian Section	Upper abdominal pain and nausea	400 g of hemorrhage	Computed tomography showed a 37-mm SAA associated with copious adjacent fluid	Splenectomy
Chenhong Wang, et al. [2]	2011	30, Primigravida	Emergency Cesarian Section	Acute onset of severe, constant left upper abdominal pain	Acute hypotension	Examination revealed diffuse abdominal tenderness with rebounding and guarding	Splenectomy
Chirag Sharma, et al. [3]	2023	25, Gravida 4, Para 3	Vaginal Delivery	Severe chest and abdominal pain	Tachycardia and hypotension	Emergent ultrasonography revealed a spleen with an irregular contour and some hypoechoic areas. Free fluid with moving internal echoes was observed in the peri-hepatic region, peri-splenic region, hepatorenal pouch, bilateral paracolic gutters, and pelvis. A contrast-enhanced computed tomography scan confirmed splenic rupture and significant hemoperitoneum	Splenectomy
Athula Kaluarachchi, et al. [5]	1995	21, Primigravida	Induced Vaginal Delivery	Acute abdominal pain and vaginal bleeding	Tachycardia	Ultrasonography of the abdomen showed infra-diaphragmatic fluid collection and intraperitoneal fluid	Splenectomy

## Discussion

Spontaneous splenic rupture during or after delivery in the absence of trauma is a rare complication that happens mostly in the third trimester or postpartum period. The classical presentation of spontaneous splenic rupture is similar to free intra-abdominal hemorrhage, which includes left shoulder pain, abdominal pain, and shock. Among the 89 reported cases of splenic rupture during pregnancy, only 2.2% were recorded to occur spontaneously after delivery. Spontaneous rupture of the spleen is a diagnosis of exclusion after any systemic diseases, prior trauma, or abnormal findings in the examination are excluded. Similarly, the splenic parenchyma, vasculature, and capsule should have normal gross and histological features [6]. The management of splenic rupture depends upon factors such as the extent of the injury, the patient’s clinical presentation, and the underlying pathology. The main established treatment protocol is emergency splenectomy. However, patients who cannot undergo surgery should be evaluated for nonoperative management such as hospital stay, splenic angiography, and splenic angioembolization under strict criteria that must be met [7]. The criteria include the absence of peritoneal distress signs or any abdominal injury that requires surgery, stable hemodynamics, absence of preexisting splenic disease, and an age below 55 years with a low-grade injury and minimal hemoperitoneum. Moreover, there is another option for treatment such as splenic artery angiography followed by embolization which showed an 85% succession rate [3]. In our case, the patient presented with dizziness, abdominal pain, and loss of consciousness after eight hours of delivery. An urgent pelvic abdominal ultrasound was ordered revealing a marked amount of free intraperitoneal turbid fluid collection as well as a large peri-splenic hematoma (15x8 cm) under the left hemidiaphragm around the spleen. An open laparotomy was performed and splenectomy was done. A large biopsy of the spleen was sent for histopathology, demonstrating a congestive red pulp with no evidence of malignancy.

## Conclusions

This is a case report on a rare incident of spontaneous splenic rupture following a singleton spontaneous vaginal delivery. Despite a lack of traumatic history or systemic disease, the patient developed this life-threatening complication. The prompt recognition of her deteriorating clinical state and the decision to proceed with an emergency exploratory laparotomy were critical in managing the situation. The performed splenectomy and subsequent histopathological examination revealed no evidence of malignancy, confirming the diagnosis of spontaneous splenic rupture. The case underscores the importance of maintaining a high index of suspicion for abdominal complications post-delivery, even in the absence of traumatic or predisposing factors. Clinicians should be aware of such rare complications and consider them in differential diagnoses to ensure prompt and effective treatment.
